# Gold nanorods as biocompatible nano-agents for the enhanced photothermal therapy in skin disorders

**DOI:** 10.7555/JBR.38.20240119

**Published:** 2024-10-08

**Authors:** Yamei Gao, Shaohu Huo, Chao Chen, Shiyu Du, Ruiyuan Xia, Jian Liu, Dandan Chen, Ziyue Diao, Xin Han, Zhiqiang Yin

**Affiliations:** 1 Department of Dermatology, the First Affiliated Hospital of Nanjing Medical University, Nanjing, Jiangsu 210029, China; 2 Department of Pediatrics, the First Affiliated Hospital of Anhui Medical University, Hefei, Anhui 230022, China; 3 State Key Laboratory on Technologies for Chinese Medicine Pharmaceutical Process Control and Intelligent Manufacture, School of Medicine, Nanjing University of Chinese Medicine, Nanjing, Jiangsu 210046, China; 4 College of Engineering and Applied Sciences, State Key Laboratory of Analytical Chemistry for Life Science, Nanjing University, Nanjing, Jiangsu 210008, China

**Keywords:** gold nanorods, photothermal therapy, skin tumors, wound healing, skin infections, inflammatory disease

## Abstract

Rod-shaped gold nanomaterials, known as gold nanorods (GNRs), may undergo specific surface modification, because of their straightforward surface chemistry. This feature makes them appropriate for use as functional and biocompatible nano-formulations. By optimizing the absorption of longitudinally localized surface plasmon resonance in the near-infrared region, which corresponds to the near-infrared bio-tissue window, GNRs with appropriate modifications may improve the results of photothermal treatment (PTT). In dermatology, potential noninvasive uses of GNRs to enhance wound healing, manage infections, combat cutaneous malignancies, and remodel skin tissues *via* PTT have attracted research attention in recent years. The review discussed the basic properties of GNRs, such as their shape, size, optical performance, photothermal efficiency, and metabolism. Then, the disadvantages of using these particles in photodynamic therapy are highlighted. Next, biological applications of GNRs-based PTT are explored in detail. Finally, the limitations and future perspectives of this research are addressed, providing a comprehensive perspective on the potential GNRs with PTT.

## Introduction

Approximately 70% of people worldwide suffer from skin illnesses^[1]^, with dermatosis being a major health issue that needs effective treatment. Conditions such as psoriasis and other inflammatory skin illnesses, wound healing, skin cancer, and skin infections are among the most common dermatological issues. In the treatment of dermatological problems, the transdermal delivery of medication is an appropriate method for topical administration^[2]^. Unfortunately, therapeutic effects of the medications that penetrate the skin to reach the epidermis or the dermis are frequently insufficient, because of the restricted permeability of pharmaceuticals into the skin, particularly the stratum corneum^[3–4]^. To address this issue, scientists have tried to treat skin conditions with nanotechnology. The goal of developing and producing nanostructures is to outperform the existing conventional formulations^[5]^.

Light can easily penetrate the skin, making it a useful tool in the treatment of skin diseases. Many ailments, such as inflammatory skin conditions, cutaneous melanoma, tissue regeneration, and infection control, are extensively treated with light technology^[6]^. Photothermal therapy (PTT) and photodynamic treatment (PDT) are prominently used in the treatment of these dermatological diseases, which have cosmetic benefits related to the improvement in the overall appearance of the affected area^[7–8]^. PDT plays a role in immune regulation, anti-inflammatory, and anti-bactericidal effects by producing reactive oxygen species. There are several challenges related to PDT, with the main ones being pain, erythema, insufficient delivery of the photosensitizer, and varying clinical response rates^[9]^. By comparison, PTT uses localized hyperthermia under optical radiation to cause the death of diseased cells or tissues^[10]^.

Depending on the required penetration depth, various types of light are employed. Near-infrared (NIR) light, which has a wavelength between 750 and 1100 nanometers, may penetrate tissues deeper than ultraviolet-visible light, and is often used to treat disorders in superficial tissues^[11–12]^. Therefore, PTT exhibits little invasiveness, remote controllability, high efficacy, and minimal drug resistance, making it a promising tool for the treatment of dermatological disorders. In recent years, the metal-based nanoparticles (MNPs) composed of pure metal nanoparticles, metal oxide nanoparticles, metal-organic frameworks, and metal nanocomposites have provided a promising approach for the photo-assisted therapies in dermatology. As photothermal enhancement agents, gold nanomaterials are more popular than other nanomaterials, because of their localized surface plasmon resonance (LSPR), easy bioconjugation, and good biocompatibility. The photothermal conversion efficiencies of various gold nanoparticles with NIR absorption capabilities, including hollow Au nanospheres, gold nanorods (GNRs), Au nano-shells, and Au nanocages, range from 22% to 103%^[13–15]^.

Among the different types of gold nanoparticles, GNRs become a prospective and low-impact alternative for topical medications because of their strong biocompatibility and functionality. GNRs, with a longitudinal LSPR in the NIR region and an ability to convert the energy of the absorbed NIR light into heat, are proper agents for both the induction of PTT and the design of the NIR radiation-responsive drug release systems. The PTT efficiency of GNRs in dermatological applications is attributed to several positive characteristics. First, the seed-mediated growth way makes it easy to create GNRs that have dimensions of around 80 nm in length and 20 nm in width^[16]^. Compared with the production process of other Au nanoparticles (NPs), this method is simpler^[17–19]^. Next, through manipulation of the aspect ratio (AR, length/width), GNRs provide an exceptional optical tunability, resulting in an improved photothermal efficiency at certain light frequencies^[20]^. Some investigators have demonstrated the use of hydroquinone to synthesize GNRs with LSPR up to 1230 nm, which have a high degree of purity, reliability, and near quantitative conversion of gold ions to metallic gold^[21]^. To control the final particle shape and size of GNRs, it has been proposed to use a binary surfactant mixture composed of hexadecyltrimethylammonium bromide (CTAB) and sodium oleate^[22–23]^. Therefore, GNRs' bioconjugation chemistry, excellent biocompatibility, and capacity to combine with other medications make them especially good metal photothermal agents^[24–26]^. PTT may also be integrated with other treatment modalities like chemotherapy and PDT, because of the chemistry involved in gold-thiol conjugation. Furthermore, GNRs penetrate deeper into the skin than other morphologies of gold nanoparticles because of their small diameters and high photothermal characteristics^[27]^. The heat induced by GNRs under NIR irradiation has the potential to alleviate the resistance of multidrug-resistant bacteria, making GNRs ideal tools for skin infection therapy. Therefore, the PTT of GNRs is more valuable in the treatment of skin diseases, enabling a non-invasive and targeted therapy.

**Figure 1:**
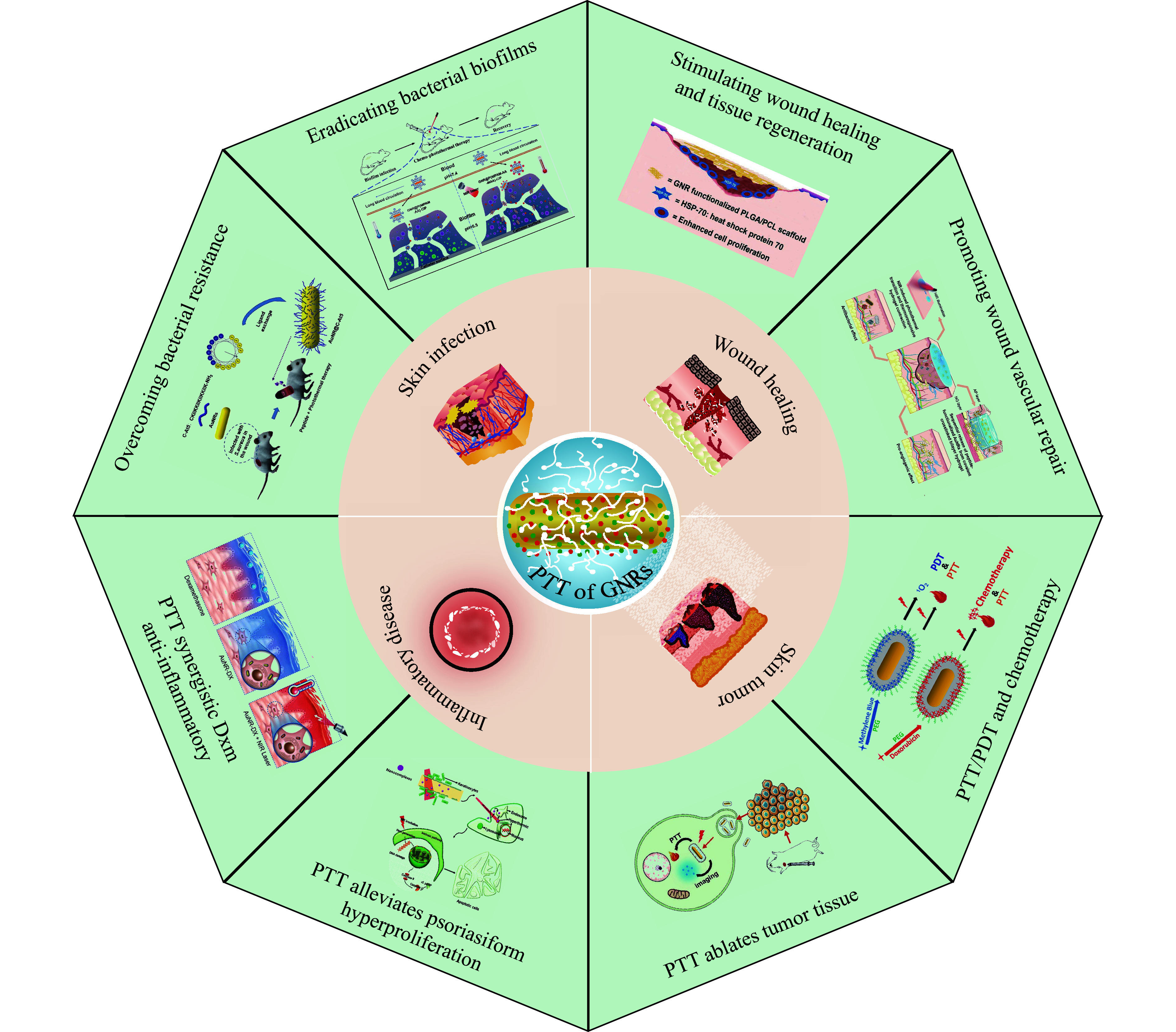
Schematic illustration of the PTT of GNR nanoparticles for dermatological applications, classified into skin infection, wound healing, skin tumor, and inflammatory disease applications. The anti-infection mechanisms of GNRs include the PTT-based overcoming of bacterial resistance and scavenging of bacterial biofilms, which were adopted from Ref.^[34–35]^ with permission of © 2022 Elsevier and American Chemical Society, respectively. The skin wound healing mechanism of GNRs includes the PTT-based proliferation of fibroblasts and endothelial cells, adapted from Ref.^[36–37]^ with permission of © 2022 American Chemical Society and © 2023 Elsevier Inc., respectively. The anti-tumor mechanisms of GNRs include the PTT-based tumor tissue ablation, which was adapted from Ref.^[38]^, and synergistic PDT therapy, which was adapted from Ref.^[39]^ with permission of © 2016 American Chemical Society. The skin anti-inflammation mechanism of GNRs includes increasing the skin penetration of drugs post-PTT and alleviating hyperproliferation, reproduced from Ref.^[40]^ with permission of © 2024 American Chemical Society and Ref.^[41]^ with permission of © 2021 Elsevier. Abbreviations: PTT, photothermal therapy; GNRs, gold nanorods; PDT, photodynamic therapy.

GNRs also have significant advantages over other photothermal agents. As for immunoregulation-associated nanofibers, the potential mechanism of interaction between their construction and cell behavior is not clearly understood, which can be evaluated in the process of complicated immunotherapeutic research in the future^[28]^. Chen *et al*^[29]^ proposed a temperature-controlling phase change fiber scaffold composed of hollow carbon fibers loaded with lauric acid as a phase change material, which might store and release any surplus heat transformed from NIR through the reversible solid-liquid transition course of the phase change material; although this nanomaterial played an important role in mild photothermal combined with chemotherapy for cancer, its range of applications was relatively narrow because of the difficulty in attaching ligands to the surface. In addition, Yang *et al*^[30]^ designed a kind of NIR-Ⅱ-triggered chitosan composite nanofiber embedded with CuSe nanoparticles, which might be applied in craniotomy of glioblastoma and simultaneously achieve a rapid hemostasis (< 8 s), an efficient superbug-killing rate (> 99%), and a complete removal of residual cancer cells; however, the synthesis method, the green electrospinning method they used, had a comparatively low productivity compared with the production efficiency of GNRs-related nanomaterials. In recent years, multimodal theranostic nanoplatforms have been extensively studied, such as phytic acid (PA)-Cu^2+^ framework/copper sulfide (Cu_2-x_S) nanocompounds (PA-Cu/Cu_2-x_S NPs) that may enhance multimodal mild-temperature photothermal therapy/chemodynamic therapy/chemotherapy by decreasing the overexpressed heat shock proteins (HSPs), and Ce6@CuS-Pt/polyethylene glycol (PEG) NPs decorated with Pt NPs as a nanozyme, showing an excellent photothermal conversion efficiency (43.08%), well singlet oxygen (^1^O_2_) production capacity, favorable physiological stability, and perfect catalytic properties^[31–32]^. However, both PA and nanozyme are expensive to manufacture and purify, complicated to operate, and sensitive to environmental conditions. Lastly, in the area of ferroelectric-photoexcited nanofiber membranes, Wang *et al*^[33]^ proposed an antibacterial nanofiber membrane [polyvinylidene fluoride/Bi_4_Ti_3_O_12_/Ti_3_C_2_T_x_ (PVDF/BTO/Ti_3_C_2_T_x_)] that exhibited an excellent photothermal effect and ferroelectric polarization, yet the pore diameter of the resulting membranes was sensitive to pressure, making it difficult to synthesize. Herein, the present review first describes the basic and optical properties of GNRs and presents their clinical application and treatment of GNRs PTT. As shown in ***Fig. 1***, we provide the insight into GNR-based PTT and its combinatorial applications in the treatment of various skin diseases, including superficial tumors, wound healing, skin injury, and skin inflammation^[34–41]^. Finally, we have summarized that GNRs should possess lower toxicity, higher yield, good monodispersity, as well as specific sizes, shapes, and aspect ratios (ARs) to achieve ideal optical properties to meet the needs for dermatosis therapy, which provides a more in-depth perspective on the potential applications of GNRs in the treatment of skin diseases.

## Basic properties of GNRs for PTT

### GNR size and shape

Micellar cationic amphiphile solutions are often used for the synthesis of GNRs. An electrochemical synthesis approach with different average ARs for GNR production in CTAB solutions was developed in 1997^[42–43]^. In 2002, Kim *et al*^[44]^ introduced a different method for creating GNRs termed photochemical synthesis, which was the process of attaching photochemically reduced gold ions to a micellar solution. Both methods enable the creation of GNRs with various ARs. Surface plasmon resonance (SPR) and substantial electron extinction are caused by the collective oscillation of GNR surface electrons when exposed to particular light wavelengths. Two bands of SPR are visible in GNRs. The first band, referred to as the transverse band, exhibits a slight extinction in visible light and is not affected by the ARs of the nanoparticles. Its wavelength range is between 500 and 550 nm. The longitudinal band, which is the second band that primarily dominates the absorption spectra of GNRs, is stronger and located in the NIR region of the spectrum^[45]^. Raising the ARs of GNRs results in a red shift and an increase in the wavelength of longitudinal band^[46]^. To meet the optical absorption criteria for PTT, this AR may be used to precisely adjust the maximum SPR and the longitudinal resonance of GNRs.

Fewer GNRs are absorbed by cells, compared with gold nanoparticles shaped like spheres (AuNSPs) or cages (AuNCs). More receptors are needed in the longitudinal direction of GNRs to start cellular absorption, because AuNSPs and AuNCs have lower ARs (approximately 1) than nanorods (approximately 2–4). CTAB is frequently used as a surfactant in the synthesis of GNRs that follow the seed growth method to stabilize the particles and prevent them from aggregating. The electropositive zeta potential of GNRs is formed by the residual CTA^+^ that covers their surface. CTA^+^ enhances cellular absorption more effectively through ionic interactions, because of the negative potential on the surface of cell membranes. The first investigation using GNRs was conducted by Du *et al*^[47]^, who used an NIR laser in an *in-vitro* PTT study, demonstrating a notable photothermal effect and the ability of GNRs to detect cancer tissues and cells. In a recent study on the PTT characteristics of GNRs of different sizes, Morales-Dalmau *et al*^[48]^ have shown that the cytotoxicity of GNRs is correlated with particle size but not as much with the precise quantity of GNRs.

**Figure 2:**
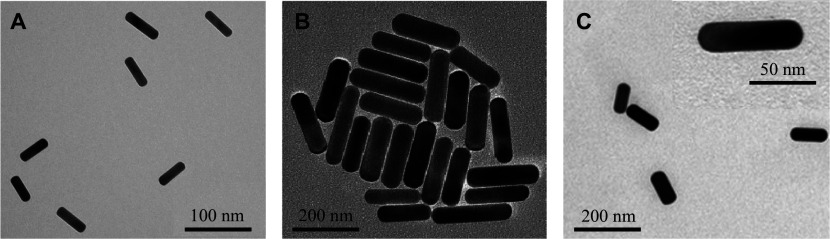
Transmission electron microscopy images of gold nanorods (GNRs). A: The GNRs had an average length of 91.2 (± 7.3) nm with widths of 26.7 (± 2.0) nm. The figure was adapted from Ref.^[52]^ with permission of © 2018 American Chemical Society. B: The average size of GNRs was 24.5 nm. The figure was adapted from Ref.^[53]^ with permission of © 2019 Elsevier Ltd. C: The diameter of the GNRs was about 25 nm and the length of the GNRs was about 75 nm. The figure was adapted from Ref.^[54]^ with permission of © 2018 American Chemical Society.

Currently, GNRs used for PTT are most likely 80 nm long and 20 nm wide, with longitudinal plasmon resonance occurring at or around 800 nm. Images captured using a scanning electron microscope revealed that the GNRs have a rod-like structure (***Fig. 2***). According to an earlier theoretical study by Jain *et al*^[49–50]^, plasmonic absorption becomes increasingly significant as the size of the nanoparticle decreases. In particular, as the GNRs increase in size, the extinction of the particles and the scattering contribution also increase. This demonstrates that when particle size decreases, the absorption/scattering ratio rises, enabling an improved photothermal conversion and a raised PTT efficiency. Manivasagan *et al*^[51]^ demonstrated that the synthesized multidentate chitosan oligosaccharide-modified GNRs (CO-GNRs) were a novel treatment for PTT of cancer cells, because of their significant absorption in the NIR spectrum, superior thermostability, and biocompatibility. Using field emission transmission electron microscopy, the average diameter and length of GNRs were measured to be 5.4 (± 3.5) nm and 24 (± 4.2) nm, respectively. Furthermore, the average diameter and length of the CO-GNRs were 6.8 (± 1.7) nm and 26 (± 3.1) nm, respectively. CO-GNRs showed a high NIR absorption peak at 838 nm. After 5 min of NIR laser irradiation at 2 W/cm^2^, the temperature of CO-GNRs rapidly rose to 52.6 ℃. The CO-GNRs showed therapeutic activity *in vitro* for the ablation of breast cancer cells with low cytotoxicity. After PTT with CO-GNRs (25 μg/mL) under laser irradiation, tumors in tumor-bearing animals were destroyed and did not recur.

### Optical properties of GNRs

Metallic particles exhibit a phenomenon known as LSPR when they are reduced to a size comparable to or smaller than the electronic mean free path, which is approximately 100 nm^[55]^. This phenomenon occurs when a plasmonic nanoscale metallic particle is exposed to electromagnetic radiation. The oscillating electromagnetic field acts on the conduction band electrons of the particles. Charge separation on the particle results from this interaction, which makes the electrons oscillate coherently with the applied field. Plasmon resonance is the greatest amplitude of oscillation at a particular frequency^[56]^. Strong absorption and scattering of the incident beam are caused by this resonance, particularly when Au-containing metallic particles are involved^[57]^. This resonance gives the GNRs their special capacity for photothermia. The metallic particle's size, shape, charge density, and element are among its physical characteristics that determine the strength of the LSPR band and the peak absorption wavelength. Gustav Mie initially accounted for these correlations in the early 1900s^[58–61]^. To achieve the optimal wavelengths for their LSPR, GNRs may be fine-tuned by modifying synthesis parameters, such as chemical concentrations of the reagent. Since only certain light wavelengths, particularly in the NIR window, are appropriate for therapy, this tunability is essential for PTT^[62]^. The range of light wavelengths that may thoroughly penetrate tissue is known as the NIR, and it extends from around 700 nm to 2500 nm, including NIR-Ⅰ (700–900 nm), NIR-Ⅱ (1000–1700 nm), NIR-Ⅲ (1500–1850 nm), and NIR -Ⅳ (2100–2300 nm)^[63]^. Because of the different penetration depths within the skin, the wavelengths between 300 and 700 nm are often used to treat superficial tissue disorders, while the wavelengths between 750 and 1100 nm penetrate further and thus are more often used for the treatment of deeper-seated tissue diseases. In PTT, NIR light with a wavelength ranging between 650 and 980 nm is preferred, while tissue absorption increases dramatically after 950 nm^[64–66]^.

**Figure 3:**
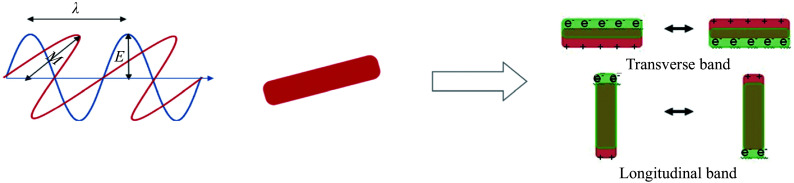
Schematic of the coherent and collective electron oscillations along the long and short axes of gold nanorods, resulting in longitudinal and transverse localized surface plasmon resonance bands, respectively. The blue line represents the electric field, and the red line represents the magnetic field. The figure is adapted from Ref.^[70]^.

The longitudinal and transverse peaks are the two distinct windows of extinction, which define the intrinsic anisotropy of GNRs. Electron oscillations along the long axis are matched by the longitudinal peak, while those along the short axis are matched by the transverse peak (***Fig. 3***). Disparate ARs may be obtained by varying the length of the GNRs, which shifts the UV-visible spectra^[67]^. The transverse absorption band remains relatively constant, while surface plasmon oscillation causes an absorption band with a longer and redshifted wavelength as the ARs increase. Gan's theory clearly explains the optical behavior underlying these phenomena^[68]^. By altering the concentration of silver nitrate in the growth fluid, the seed-mediated synthesis method allows for the adjustment of the optical range of GNRs^[45,69]^. GNRs with greater ARs are formed, when silver nitrate concentrations increase.

### Photothermal properties of GNRs

**Figure 4:**
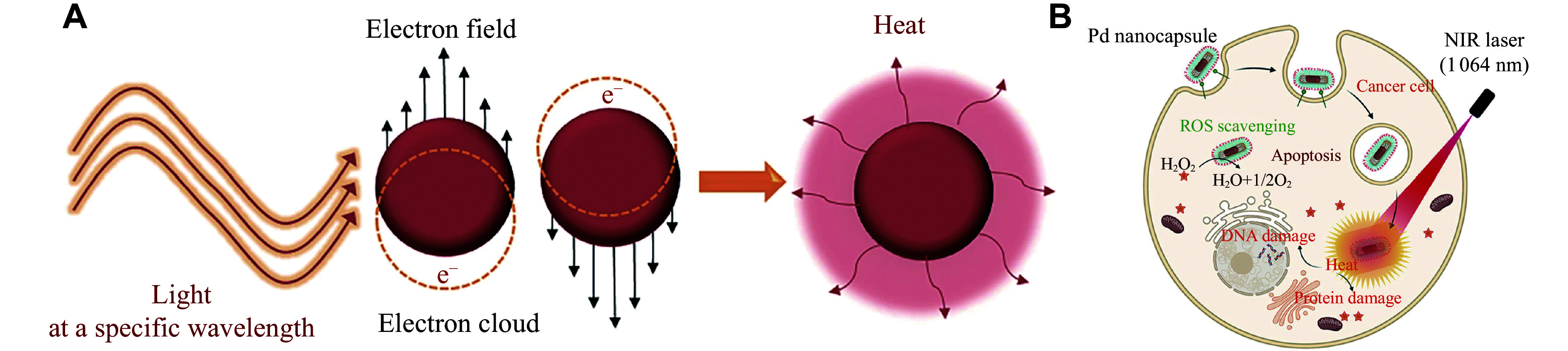
Metal-based nanoparticles convert light energy into heat by absorbing the light at a specific wavelength. A: The localized surface plasmon resonance of metal-based nanoparticles and the process of producing photothermal treatment. The figure was reproduced from Ref.^[76]^ with permission from Copyright © 2022 Controlled Release Society. B: Schematic representation of the targeted photothermal treatment therapy of nanocapsules. The figure was adapted from Ref.^[77]^ with permission from the American Chemical Society. Abbreviations: NIR, near-infrared; Pd, palladium; ROS, reactive oxygen species.

The photothermal conversion property of GNRs, which mainly depends on their concentration, shape, LSPR, *etc.*, is an important characteristic that helps in the treatment mechanism. As shown in ***Fig. 4A***, when the light at a specific wavelength is absorbed by metal-based nanoparticles, the collective oscillation of the electron cloud at the surface may convert light energy into heat for PTT. Because of this ability, GNRs may be applied to killing cells in areas exposed to NIR irradiation^[71]^. The gold nanomaterials, using a phonon-united course, convert the NIR into thermal energy, as shown in ***Fig. 4B***. Specifically, the energy from the incident wave may transmit to GNRs in the form of free electrons as soon as it has been absorbed^[72]^. The whole process of this phenomenon occurs in picoseconds, with hundreds of picoseconds spent transferring heat to the adjacent medium. In 2018, Kim *et al*^[73]^ found that GNRs exhibited the best photothermal conversion property, when the wavelength of the longitudinal plasma resonance λ matched that of the incident laser λ. On the other hand, because of the scattering capability of gold nanoparticles, the larger their size, the lower their photothermal conversion efficiency^[74]^. Furthermore, the concentration of GNRs is correlated with the photothermal conversion.

## Treatments of dermatosis by photothermal effect of GNRs

Regarding dermatological treatment, there has been an increasing interest in the photothermal effect of GNRs. Numerous studies have shown the effectiveness of various modified or coated GNRs in treating cutaneous tumors, healing wounds, preventing infections, and managing psoriasis. GNRs produce heat and eliminate over-proliferating keratinocytes and cancer cells, when subjected to light or laser light. Moreover, GNRs may function as a platform for achieving multimodality through pharmacological or gene combinations.

### Applications of GNRs in the treatment of superficial skin cancers

Malignant melanoma, squamous cell carcinoma, and basal cell carcinoma are examples of superficial skin malignancies that are both common and seriously dangerous to people's health^[75]^. Currently, the most widely used anti-cancer treatments include chemotherapy, radiation therapy, and surgical treatment. However, these treatments have certain drawbacks, including a high rate of recurrence, a poor rate of curative efficacy, significant adverse effects, and poor patient compliance. GNR-mediated phototherapy and combination treatment have emerged as replacement treatments to overcome these drawbacks. Specific details are presented in ***Table 1***.

**Table 1:** Summary of gold nanorods (GNRs)-mediated treatment of skin tumors

Materials	Evaluation models	Key findings	Ref.
Platelet-facilitated photothermal tumor therapy	*In vitro* and *in vivo* using the method of electroporation	This therapy could effectively inhibit the growth of head and neck squamous cell carcinoma.	[78]
Nanoparticles directed by ShTxB as a local treatment	Preclinical murine models and patient biopsy cells	This research showed an effective treatment for aggressive localized cancer.	[81]
Photothermal effects mediated by EGFRmAb-GNRs	In an animal model of laryngeal squamous cell carcinoma	The EGFRmAb combined with GNRs achieved a photothermal effect in the treatment of laryngeal squamous cell carcinoma.	[82]
Load the photosensitizer gold nanoparticles cage for immunogenic phototherapy of aggressive melanoma	In B16-F10 cells and in C57BL/6 female mice	This material may destroy the main cancer with NIR irradiation, and clear away the antitumor immunity to inhibit the metastasis and recurrence as well.	[83]

Lasers or illuminating light may be used to eliminate tumors or destroy cancer cells, when GNRs build up in them. A cancer treatment strategy called platelet-facilitated photothermal tumor therapy (PLT-PTT) was proposed by Rao *et al*^[78]^ in 2017. Platelets (PLTs) were used in this treatment to improve the effects of PTT by delivering photothermal agents to tumor tissues. Using the *Tgfbr1*/*Pten* conditional knockout (2cKO) mouse model, they observed that the administration of PLT-GNRs and the localizing laser irradiation effectively inhibited the growth of head and neck squamous cell carcinoma. This demonstrates the unique advantages of PLT-PTT in the management of cancer. In terms of the targeted therapy, Jin *et al*^[79]^ believed that the use of a multifunctional peptide coating mainly composed of, from the inside out, a mitochondrial targeting segment, a cathepsin B-responsive segment, and a zwitterionic antifouling segment might increase the ability of tumor recognition. Therefore, the multifunctional peptide (CC-EKEK-V-FrFKFrFK-V-GFLG-V-EKEKEKEKEKEK) was used to modify GNRs, a classical photothermal agent, to construct a multifunctional therapeutic nanoplatform (GNR@FrFK-GFLG-EK) for PTT and mitochondrial targeting. Meanwhile, Ding *et al*^[80]^ proposed a kind of tumor microenvironment-responsive multifunctional peptide (Tat-R-EK) coated ultrasmall gold nanoparticles that might effectively solve the short circulation half-life, non-specific distribution, slow clearance, and low radio-sensitizing effect. Additionally, Navarro-Palomares *et al*^[81]^ described the effective conjugation of a high-affinity protein-ligand functional group (Shiga Toxin-B; ShTxB) with GNR in 2022. They discovered that the ShTxB-functionalized GNRs were efficiently retrotranslocated to cytoplasms of globotriaosylceramide (GB3)-positive cells. Further laser irradiation at the SPR wavelength of the longitudinally positioned GNRs stimulated the death of the targeted tumor cells. Specifically, more than half of GB3-positive neoplastic cells that contained GNR@SiO_2_@ShTxB were eliminated after just 3 min of NIR radiation, while a longer laser exposure (10 min) killed 80% of these cells^[81]^. In the same year, Hai *et al*^[82]^ showed that the conjugates of epidermal growth factor receptor monoclonal antibody (EGFRmAb) and GNRs selectively entered squamous cancer cells, and exerted the photothermal effect, causing cancer cell death and inhibiting the proliferation of laryngeal squamous cell carcinoma cells. Furthermore, Xie *et al*^[83]^ designed a multifunctional nanoplatform MLI-AuNCs, in which additive monophosphoryl lipid A and sensitizer indocyanine green were sheltered and co-transferred to the tumors by the thermosensitive lipid-enveloped AuNCs, which exhibited remarkable NIR-triggered cell-killing ability.

### The use of GNRs for promoting wound healing and repairing skin defects

Because wound healing has such a large impact on human health, the interest in this area has lately surged. Wounds provide a favorable environment for microbial growth, thus there is an increased risk of skin infection during the healing process^[84]^. Creating efficient treatment plans that encourage wound healing, restore skin function, and avert complications is crucial to solving this problem^[85]^. GNRs, which are detailed in ***Table 2***, have attracted a significant interest as a possible wound-repairing agent and have demonstrated promise in some medical applications. In one study of human skin fibroblasts, the investigators evaluated the cytotoxicity, cellular uptake, and wound-healing properties of several ligand-modified GNRs, among which GNRs modified with neutral and cationic PEG exhibited low levels of cytotoxicity and cellular internalization, while GNRs coated with anionic ligands and bovine serum albumin (BSA) demonstrated a strong cytotoxicity and cellular uptake in human skin fibroblasts^[86]^. The results of a cell scraping experiment showed that the rate of wound healing significantly increased after incubating neutral cationic PEG-modified GNRs and anion-modified GNRs with the scraped human skin fibroblasts for 24 h, compared with the control group. On the other hand, the injured fibroblasts exposed to BSA-GNRs showed a significant delay in wound healing rate and the release of the inflammatory cytokine interleukin 1β. The surface neutral or cationic modification of GNRs is directly correlated with their ability to increase cellular internalization, and the anionic ligands are harmful to skin cells. Controlling local and systemic elements, including inflammation, proliferation, and maturation, during the healing process is essential to the effectiveness of wound healing. Nanda *et al*^[36]^ developed a polylactic glycolic acid (PLGA)/polycaprolactone scaffold containing GNRs to accelerate wound healing. The GNR-containing scaffold increased the local temperature to 40 ℃ at the wound of mice following laser irradiation, which further upregulated the expression of HSP70 and promoted the wound healing. In 2023, Singh *et al*^[87]^ developed a nanoplatform with a core polymer, poly(N-isopropylacrylamide), combined with GNRs, which might control and safely deliver drugs to wound sites, thereby promoting targeted wound healing. Furthermore, in the same year, inspired by nanometer array control of cell behavior, Wang *et al*^[88]^ designed spiky gold-palladium heterostructured nanoparticles (AuPd SHs) with topographical surface architectures. These nanoparticles were capable of promoting multistage wound healing in a programmable manner. Considering that PTT may provide conditions for the on-demand delivery of bio-active molecules, Nakielski *et al*^[89]^ designed a plasma hydrogel that might induce structural changes in the hydrogel by activating GNRs with NIR light to generate heat, leading to controlled drug release.

**Table 2:** Summary of gold nanorods (GNRs)-mediated treatment of wound healing

Materials	Evaluation models	Key findings	Ref.
GNRs decorated with different ligands	On human dermal fibroblasts	PEGylated and charged-GNR accelerated the wound healing rate of scratched human dermal fibroblasts *in vitro*.	[86]
GNR-incorporated poly(lactic-co-glycolic acid) (PLGA)/poly(caprolactone) (PCL)	The GNR-incorporated scaffold attached to the wound of mice	The GNR-incorporated scaffold and timely control of HSP70 expression may be used as a promising wound-healing strategy.	[36]
Smart delivery platform using core-shell nanofibers for sequential drug release in wound healing	*In vitro* experiments	The authors provided a versatile platform for controlled and safe drug delivery to wound sites, which could be applied to the treatment of other topical diseases.	[87]
Spiky surface topography of heterostructured nanoparticles for programmable acceleration of multistage wound healing	*In vitro* and in the wounding model in mice	Spiky AuPd SHs with simple composition and compact structures exhibit hierarchical acceleration in multiple stages of wound healing.	[88]

### GNRs for combating skin infections

As a significant part of dermatologic illnesses, skin infections caused by bacteria, fungi, and viruses may result in life-threatening health problems. High dosages of antibiotics are frequently needed to provide a decent therapeutic effect, because they are ineffective in reaching the infection site^[90]^. On the other hand, using large dosages of antibiotics raises the possibility of microbial resistance, because microbiota growth is strongly influenced by the dosage and duration of treatment^[91]^. To solve this issue, a viable substitute known as GNRs-mediated PTT has been created. ***Table 3*** provides a concise and straightforward summary of our work. In 2021, Niu *et al*^[92]^ developed a nanoconjugate with GNRs decorated with pH-induced charge-switchable tertiary amine groups with bacteriophilic phenylboronic acid molecules to target specific bacterial cells and destroy them using photothermal ablation. GNRs produced heat in response to infrared light stimulation, thereby efficiently destroying the target bacterial cells and accelerating the healing of wounds in diabetic mice. Meanwhile, Sheng *et al*^[93]^ designed infection microenvironment-responsive multifunctional peptide-coated GNRs for bimodal antibacterial applications, which had both targeting capability and biodegradability. The same targeting issues of GNRs were addressed by Chen *et al*^[94]^, who devised a combination of GNRs with antimicrobial peptides that had a lower tendency than traditional antibiotics to engender drug resistance in bacteria. Furthermore, in 2022, Zhang *et al*^[95]^ functionalized GNRs with metal-phenolic networks (MPNs) *via* a coordination reaction, yielding GNR@MPNs, a tunable biocompatible photothermal nano-sterilization product. These bioactive GNR@MPNs had a high photothermal conversion efficiency that eliminated 99.8% of *Escherichia* *coli* and 98.6% of *Staphylococcus* *aureus* with a comparatively low effective dose. Lv *et al*^[96]^ presented the molybdenum disulfide (MoS_2_)-GNRs-apt in 2023, and found that the incorporation of MoS_2_ nanosheets significantly enhanced the biocompatibility of GNRs. The antimicrobial property of MoS_2_-GNRs-apt was much better than that of non-targeted PTT, and they might precisely defeat multidrug-resistant *Pseudomonas* *aeruginosa* and effectively decrease superfluous M1 inflammatory macrophages to promote infected wound healing. In 2024, Hong *et al*^[97]^ proposed an innovative nanomaterial-based therapeutic approach (PAu@C/B) with photo-triggered antimicrobial and anti-inflammatory activities, which improved infectious skin tissue regeneration. To promote infectious skin regeneration, this approach not only exerted an antibacterial effect by uniting mild PTT and PDT, but also inhibited inflammation and promoted growth factor production, collagen deposition, and angiogenesis by controlling drug release.

**Table 3:** Summary of gold nanorods (GNRs)-mediated treatment of skin infections

Materials	Evaluation models	Key findings	Ref.
GNR decorated with bacterial affinitive molecules phenylboronic acid and pH-induced charge-convertible tertiary-amine group for chronic wounds	In bacterial cells and diabetic rats	Target specific bacterial cells and destroy them using PTT.	[92]
A selective therapeutic nanorod (MoS2-GNRs-apt) based on molybdenum disulfide (MoS2) nanosheets coated GNRs	In Pseudomonas aeruginosa (MRPA)-infected wound murine model	This molecular therapeutic strategy displays great potential as a prospective antimicrobial treatment for MRPA infections.	[96]
Metal-phenolic networks (MPNs) were employed to functionalize GNRs	A mice model infected by methicillin-resistant *S. aureus*	The GNRs@MPNs may precisely defeat MRPA bacteria and effectively decrease superfluous M1 inflammatory macrophages.	[95]
Gold-based nanoflower composite with dual drug incorporation	*In vitro* and in animal models of subcutaneous abscess and skin wound infected with drug-resistant bacteria	It showed good biocompatibility and may improve infectious skin regeneration by its antibacterial/anti-inflammatory effect.	[97]

### The use of GNRs for treating inflammatory disease

Two to five percent of people worldwide suffer from psoriasis, a chronic immune-mediated skin condition characterized by scaling, erythema, and thickness^[98]^. The increased inflammatory response, overproduction of pro-inflammatory cytokines, and excessive keratinocyte proliferation are the primary pathogenic features of psoriasis^[99–100]^. As a result, psoriasis treatment plans aim to lower inflammation and halt keratinocyte proliferation. GNRs have emerged as a potentially effective treatment option for psoriasis in recent times. Studies have shown that they may cause apoptosis by high temperatures when exposed to NIR light, as shown in ***Table 4***.

**Table 4:** Summary of gold nanorods (GNRs)-mediated treatment of inflammatory disease

Materials	Evaluation models	Key findings	Ref.
Silver and gold nanoparticles complexed with *Cornus* *mas* extract	*In vivo* and *in vitro* experiments	This technology provided an efficient tool for modern psoriasis therapy, circumventing immunosuppression-related side effects of biologicals.	[101]
Gold nanorods (GNRs) and isatin were loaded into a poly (lactic-co-glycolic acid) matrix to form the nanocomplexes	Live/dead cell assay and *in vivo* psoriasiform murine model	The as-prepared nanocomplexes allowed for hyperthermia-induced apoptosis of keratinocytes, and served as a promising therapy against hyperproliferation.	[41]
A sub-15 nm nanoparticle containing a 3 nm gold core and a shell of 1 000 Da polyethylene glycol strands modified with 30% octadecyl chains	*In vitro* and in psoriasis mice	This self-therapeutic nanoparticle might be topically delivered to epidermal keratinocytes to prevent and treat psoriasis.	[102]
A novel strategy to conjugate gold nanorod and dexamethasone	In imiquimod-induced mouse models and HaCaT cells	The study highlighted the GNRs and dexamethasone-conjugated enhancement drugs through the potential of the dermis.	[40]

For example, Crisan *et al*^[101]^ have demonstrated the superior anti-inflammatory properties of polyphenol-rich *Cornus* *mas* extract against psoriasis. At both the cellular and molecular levels, GNR and *Cornus* *mas* extract (Au NPs-CM) worked together to control psoriasis inflammation. Ag and Au NPs-CM is a nanoparticle-based technology that offers an alternative to biological therapies for psoriasis by circumventing the immunosuppressive adverse effects. In 2021, Nirmal *et al*^[41]^ investigated the use of photothermal nano-systems in the management of psoriasis, suggesting a technique to make GNR-containing nanocomposites with isatin, an anti-inflammatory medication, embedded in a PLGA matrix, where these GNR-loaded nanoparticles, when exposed to NIR radiation with an intensity of 0.42 W/cm^2^, might convert the NIR radiation into heat, increasing the temperature by 10 ℃; combined with the NIR radiation, the nanocomplexes internalized by keratinocytes induced apoptosis through the caspase and poly-ADP-ribose polymerase pathways. In 2022, Han *et al*^[102]^ reported that the combination of nanocomplexes and NIR light inhibited neutrophil infiltration and epidermal hyperplasia in an *in vivo* mouse model of psoriasis. In this study, they proposed a nanoparticle containing a 3-nm gold core and a shell of PEG chains (1000 Da) modified with 30% octadecyl chains, which was smaller than 15 nm and might penetrate the stratum corneum and enter keratinocytes specifically. Co-administration of imiquimod and nanoparticles inhibited the growth of psoriasis and downregulated genes involved in the interleukin-17 signaling pathway that contributed to inflammation and epidermal hyperplasia. In 2024, Kim *et al*^[40]^ employed a novel strategy to conjugate GNRs and dexamethasone (a primary corticosteroid for treating psoriasis) to address the limited skin permeation of dexamethasone, thereby mitigating its documented adverse effects. Moreover, in an *in vivo* psoriasiform murine model, Fang *et al*^[103]^ found that the GNR nanocomplexes remained in the skin for at least five days, and that the nanocomposites produced a negligible toxicity in the skin of healthy mice, suggesting that GNRs may act as fillers for the treatment of skin injury and wounds.

## Limitations of GNRs for PTT

### Biocompatibility and cytotoxicity of GNRs

**Figure 5:**
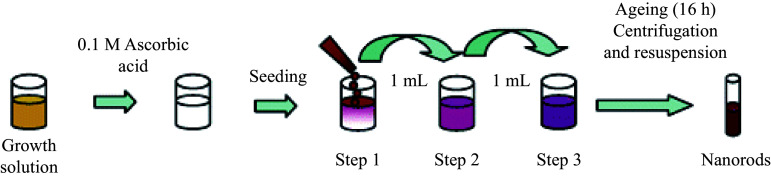
General synthetic method for the generation of gold nanorods. The figure was adapted from Ref.^[112]^ with permission of © 2004 American Chemical Society.

As shown in ***Fig. 5***, the general method for synthesizing GNRs is a seed-growth method. In the synthesis process, CTAB acts as a cationic surfactant and significantly increases the productivity of GNRs. GNRs will aggregate irreversibly without a certain number of ligands on their surface. However, the biological toxicity of GNRs mainly comes from the positive charge of CTAB, resulting in necrocytosis^[104]^. Commonly used methods include exchanging CTAB with chemisorptive agents, such as thiols, phospholipids, and citrate, or coating the surface with other solid-state materials^[105–107]^. The biocompatibility of GNRs requires a comprehensive understanding of their biomedical effects to recognize any potential toxicity matters^[107–109]^. The specific risks of cytotoxicity related to the application of GNRs remain unclear, because few investigators have addressed this problem in previous studies, when they emphasize their good biocompatibility. On the contrary, some believe that GNRs themselves do exhibit some cytotoxicity^[110]^. Various factors, such as the size or shape of the nanoparticles and their surface chemistry, may result in the toxicity of GNRs^[111]^.

Effective endocytosis, relying on many physicochemical and biological factors, is considerably important in the degradation process of GNRs *in vivo*, and some factors may affect the endocytosis of GNRs^[113]^. First, the size and AR of rod shaped-gold nanoparticles play an important role in cellular uptake. Neither oversized nor undersized GNRs may result in efficient uptake, because undersized nanoparticles are usually too small to bind ligands with receptors, and oversized GNRs diffuse slowly^[114]^. According to the literature, GNRs with a size range between 20 and 50 nm may achieve a good endocytic efficiency, with the optimum cellular uptake occurring at 50 nm^[114–116]^. Meanwhile, the efficiency of uptake increases along with the decrease in AR because of the mechanisms of endocytosis of GNRs: receptor-mediated endocytosis and caveolae-dependent pinocytosis^[113–115,117–118]^. Surface chemistry also plays an important role in the interaction between nanorods and cells. Additionally, several other factors, such as cell types, substrate constitutions, brooding time, *etc.*, may influence cellular uptake^[118–122]^. In the metabolism process of GNRs, although gold nanoparticles were considered nontoxic, recent experiments *in vivo* and *in vitro* have revealed potential health implications for the use of GNRs^[123]^. For example, GNRs may induce cellular toxicity and hepatotoxicity in mice, cross the blood-testis barrier, and deposit in the testes, which affects male reproduction^[124]^. Xu *et al*^[125]^ proposed that the systemic toxicity of GNRs was mainly affected by their shape, time, and dose; moreover, the results indicated that short GNRs triggered a more serious disorder of cell metabolism, and high concentrations of GNRs caused more significant toxicity. The main toxicity mechanism of GNRs on HepG2 cells was the accumulation of GNRs in cell sub-organs, such as mitochondria, affecting tricarboxylic acid cyclic metabolism and thereby reducing energy production. Hu *et al*^[126]^ also showed that the photothermal process of GNRs might induce cytotoxicity by affecting pyruvate and glutamate synthesis, normal choline metabolism, and ultimately apoptosis. In recent years, many studies have proposed improving metabolic effects on cancer and non-cancer cells through surface modification of GNRs. For example, positively charged polyethylenimine (PEI)-GNRs showed the highest antitumor effects on A549 cancer cells because of their energy metabolism, choline metabolism, hexosamine biosynthesis pathway, and oxidative stress-related metabolism^[127]^. Phospholipid-GNRs significantly decreased the levels of energy metabolism, resulting in dysfunction in the tricarboxylic acid cycle, a reduction in glycolytic activity, an imbalance of the redox state, and the proliferation of cancer cells^[128]^.

### Diffusion of GNRs within deep skin tissues

Although GNRs theoretically show significant light absorption, Ali *et al*^[129]^ found that their distribution within human cells upon injection was random. The study reported that PEGylated GNRs primarily accumulated in the liver and spleen of mice, where they might stay for up to 15 months without becoming hazardous. It is uncertain, therefore, if these GNRs are safe for use in clinical settings, particularly in cases when total particle removal is not possible. Drug-loaded GNRs are less therapeutically effective than drug-free ones because of their size, which prevents tissue diffusion^[130]^. Li *et al*^[131]^ demonstrated that smaller GNRs (7 nm in diameter) were more effectively eliminated *in vivo* than larger GNRs (14 nm in diameter); besides, other attributes required for biomedical applications were also present in these smaller GNRs, including high cellular uptake, minimal cytotoxicity, and photothermal efficiency; thus, one potential tactic to increase the removal rate might be to change the size of the GNRs. Like in earlier research, we found that GNR size mattered because smaller GNRs were more likely to permeate and accumulate beneath the epidermis. The poor concentration of GNRs at the target site is another issue that may compromise the effectiveness of treatment. The active targeted release of GNRs may be accomplished by using particular ligands, including peptides, antibodies, and aptamers, to get around this issue. It has been demonstrated that this strategy works very well to increase the uptake of particles by tumor tissues. For instance, compared with non-targeting GNRs, GNRs functionalized with GNR-targeting peptides [Au25(Capt)18] have superior tumor penetration and accumulation capabilities^[132]^.

## Conclusions

This review discusses the use of GNRs in various dermopathy therapies in recent years. To achieve a better therapeutic effect, GNRs must possess the characteristics such as hypotoxicity, high productivity, good dispersity, and standard particle sizes, shapes, and ARs to ensure perfect photothermal conversion performance. Meanwhile, we have summarized the common synthetic methods, basic properties, and biological applications of GNRs in PTT. Among these methods, the seed-mediated growth method is widely used, because of its easy AR control. Numerous investigators have enhanced the properties of GNRs by fine-tuning synthesis conditions. The toxicity of GNRs can be reduced by highly centrifugal cleaning and removing or masking the native CTAB surfactant on the nanorod surface^[133]^. Of course, other properties of GNRs may also change as the synthetic parameters are adjusted.

However, investigators hold different opinions on the optimal shape and size of gold nanoparticles. Gold nanoparticles with a rodlike structure possess a better optical property with the SPR effect, compared with other shapes. As for the size of GNRs, the thermal production efficiency, body residues, and skin penetration decrease, as their size increases. Nevertheless, smaller GNRs may present higher cytotoxicity because of their large specific surface area, which leads to deposition on healthy organs and tissues. Therefore, the small size of GNRs may achieve better treatment effects, but also lead to toxic deposits in normal tissues^[134]^. To solve this problem, some investigators have sought innovative methods to synthesize GNRs, because traditional methods may not achieve the designed size of GNRs, leading to low production and poor dispersion^[135]^.

To obtain better therapeutic effects, some key points need to be achieved, such as increasing skin retention time, enhancing targeting to disease cells, increasing endocytosis, reducing cytotoxicity, improving photothermal conversion efficiency, and eliminating excessive proliferation cells, among others. Furthermore, different reaction conditions may lead to undesirable results, as seen in research on the influence of ectochemiscal elements on endocytosis^[136]^. Additionally, many studies have not been performed *in vivo*, suggesting that physical simulation experiments may lack comprehensive representation^[137]^. In addition, the high endocytosis of GNRs often achieves a good therapeutic effect, it also has toxicity. Literature suggests that GNRs with surface ligands may be better phagocytosed by the cells than naked GNRs, which may benefit PTT applications. Furthermore, more animal models should be developed to simulate human physical structure and behaviors to predict the transport, distribution, excretion, and degradation of GNRs *in vivo*. GNRs possess several advantageous properties, such as good biocompatibility, high photo-thermal transformation efficiency, excellent optical capabilities, easy synthesis, easy surface modification, and controllable size and AR. Traditional GNRs may only provide single-mode biomedical imaging or cancer therapy mainly because of the limited functionalization. Therefore, advanced structural control strategies are necessary for the preparation and synthesis of GNR nanoparticles to meet actual clinical needs. The following modification strategies have been applied to the surfaces of GNRs: 1) metal deposition at the tips of GNRs or metal coating on the surface of GNRs; 2) a second class of GNRs that are coated with other organic functional shells; and 3) various surface functional groups and compounds that not only improve their biocompatibility *in vivo*, but also enable GNRs with a series of amazing biomedical capabilities including photoacoustic, X-ray therapy, PTT, PDT, and chemotherapy for cancer treatment^[123,138–139]^. In addition, the excretion and biocompatibility of GNRs are critical factors. Therefore, more GNRs with excellent biocompatibility should be developed for clinical studies. Thus, we surely believe that nano-composites based on GNRs will have a potential application in the treatment of skin diseases.

However, there are many limitations to the biological application of GNRs, including long-term stability, biological toxicity, *in vivo* distribution, and final fate after administration. GNRs are toxic in their own right, because they are taken up by Kupffer cells and accumulate in the liver after circulating *in vivo*, causing organ damage by inducing cell mitochondrial damage and lipid peroxidation^[140]^. In addition, surface modifications may affect cytotoxicity, such as hemolysis induced by positively charged CTAB-modified GNRs^[141]^. The PTT and NIR responsive drug delivery systems benefit from the NIR GNR LSPR sensor's ability to transform the absorbed NIR light into heat. The use of GNR-based PTT in dermatological therapy has grown in popularity in recent years. The straightforward surface chemistry of GNRs facilitates their modification with a wide range of reagents, including peptides, antibodies, and aptamers. Furthermore, GNRs may work as multipurpose platforms that may combine multiple drugs to create complex therapeutic systems. In addition, the high endocytosis of GNRs often achieves a good therapeutic effect yet has toxicity, but GNRs with surface ligands may be better phagocytosed by the cells, which may be used in GNRs for PTT applications. Numerous drug release mechanisms have been employed, including dose-dependent release, enzymatic release, light/thermal-triggered release, and pH-sensitivity release. Through the manipulation of surface chemistry and drug release mechanisms, GNRs may supply a range of functional drugs for chemotherapy-paired PTT.

Although GNRs show some promise in the treatment of dermatosis, there are several issues to consider: they may be cytotoxic, take a while to biodistribute, and not penetrate as deeply into the skin as free drugs can. Therefore, new approaches need to be developed to increase treatment efficacy and target accumulation efficiency. Furthermore, before being used on patients, further comprehensive research is needed on the subcutaneous penetration processes of GNRs and the results of post-treatment. Thus, there is still a long way to go before these therapies are used in clinical settings.
